# Optogenetic recruitment of spinal reflex pathways from large‐diameter primary afferents in non‐transgenic rats transduced with AAV9/Channelrhodopsin 2

**DOI:** 10.1113/JP278292

**Published:** 2019-08-28

**Authors:** Shinji Kubota, Wupuer Sidikejiang, Moeko Kudo, Ken‐ichi Inoue, Tatsuya Umeda, Masahiko Takada, Kazuhiko Seki

**Affiliations:** ^1^ Department of Neurophysiology National Institute of Neuroscience National Center of Neurology and Psychiatry Kodaira Tokyo Japan; ^2^ Research Fellow of the Japan Society for the Promotion of Science Chiyoda‐ku Tokyo Japan; ^3^ Systems Neuroscience Section Department of Neuroscience Primate Research Institute Kyoto University Inuyama Aichi Japan; ^4^ PRESTO Japan Science and Technology Agency Kawaguchi Saitama Japan

**Keywords:** Adeno‐associated virus 9, Dorsal root ganglion neurons, Aα/β‐fibres, Channelrhodopsin 2, Spinal reflex pathway, Optogenetics

## Abstract

**Key points:**

- We demonstrated optical activation of primary somatosensory afferents with high selectivity to fast‐conducting fibres by means of adeno‐associated virus 9 (AAV9)‐mediated gene transduction in dorsal root ganglion (DRG) neurons.
- AVV9 expressing green fluorescent protein showed high selectivity and transduction efficiency for fast‐conducting, large‐sized DRG neurons.
- Compared with conventional electrical stimulation, optically elicited volleys in primary afferents had higher sensitivity with stimulus amplitude, but lower sensitivity with stimulus frequency.
- Optically elicited dorsal root volleys activated postsynaptic neurons in the segmental spinal pathway.
- This proposed technique will help establish the causal relationships between somatosensory afferent inputs and neural responses in the CNS as well as behavioural outcomes in higher mammals where transgenic animals are not available.

**Abstract:**

Previously, fundamental structures and their mode of action in the spinal reflex circuit were determined by confirming their input–output relationship using electrophysiological techniques. In those experiments, the electrical stimulation of afferent fibres was used as a core element to identify different types of reflex pathways; however, a major disadvantage of this technique is its non‐selectivity. In this study, we investigated the selective activation of large‐diameter afferents by optogenetics combined with a virus vector transduction technique (injection via the sciatic nerve) in non‐transgenic male Jcl:Wistar rats. We found that green fluorescent protein gene transduction of rat dorsal root ganglion (DRG) neurons with a preference for medium‐to‐large‐sized cells was achieved using the adeno‐associated virus 9 (AAV9) vector compared with the AAV6 vector (*P* = 0.021). Furthermore, the optical stimulation of Channelrhodopsin 2 (ChR2)‐expressing DRG neurons (transduced by AAV9) produced compound action potentials in afferent nerves originating from fast‐conducting nerve fibres. We also confirmed that physiological responses to different stimulus amplitudes were comparable between optogenetic and electrophysiological activation. However, compared with electrically elicited responses, the optically elicited responses had lower sensitivity with stimulus frequency. Finally, we showed that afferent volleys evoked by optical stimulation were sufficient to activate postsynaptic neurons in the spinal reflex arc. These results provide new ways for understanding the role of sensory afferent input to the central nervous system regarding behavioural control, especially when genetically manipulated animals are not available, such as higher mammals including non‐human primates.

## Key points

- We demonstrated optical activation of primary somatosensory afferents with high selectivity to fast‐conducting fibres by means of adeno‐associated virus 9 (AAV9)‐mediated gene transduction in dorsal root ganglion (DRG) neurons.
- AVV9 expressing green fluorescent protein showed high selectivity and transduction efficiency for fast‐conducting, large‐sized DRG neurons.
- Compared with conventional electrical stimulation, optically elicited volleys in primary afferents had higher sensitivity with stimulus amplitude, but lower sensitivity with stimulus frequency.
- Optically elicited dorsal root volleys activated postsynaptic neurons in the segmental spinal pathway.
- This proposed technique will help establish the causal relationships between somatosensory afferent inputs and neural responses in the CNS as well as behavioural outcomes in higher mammals where transgenic animals are not available.

## Introduction

Optogenetics describes the combination of genetic and optical methods (Zemelman *et al*. 2002; Boyden *et al*. 2005) to manipulate the activity of neuronal networks with the aim of determining their roles in the control of specific behaviours. This technique uses the gene expression of light‐activated microbial proteins, opsins, in target neuronal (Boyden *et al*. 2005) or non‐neuronal cell populations (Gradinaru *et al*. 2009). Depending on the opsin used, a wide variety of light‐induced changes in cell activity are possible (Kim *et al*. 2017). A hallmark of optogenetics is its high temporal and spatial resolution in manipulating real‐time neuronal activity (Deisseroth, 2011). Neuroscience studies have taken advantage of this technology to determine the causal relationships of neuronal activity and their behavioural relevance by manipulating neuronal activity and monitoring consequential behavioural changes in awake, behaving animals (Adamantidis *et al*. 2007; Gradinaru *et al*. 2009). To date, most optogenetic applications have focused on the central nervous system (CNS) (Galvan *et al*. 2017; Rost *et al*. 2017) and it has rarely been applied to the peripheral nervous system (PNS). Although the application of optogenetics to the PNS has clear advantages for both therapeutic (Llewellyn *et al*. 2010; Daou *et al*. 2013; Liske *et al*. 2013; Copits *et al*. 2016; Iyer *et al*. 2016; Srinivasan *et al*. 2018) and physiological (Park *et al*. 2016; Abe & Yawo, 2017; Arcourt *et al*. 2017) perspectives, a number of technical difficulties have precluded the application (Montgomery *et al*. 2016).

Among them, a major difficulty is the heterogeneous nature of primary sensory neurons (dorsal root ganglion (DRG) neurons), which are composed of different classes of neurons, each of which has a distinct sensory modality such as nociception and proprioception (Hammond *et al*. 2004; Fang *et al*. 2005; Ruscheweyh *et al*. 2007; Usoskin *et al*. 2015; Li *et al*. 2016; Xie *et al*. 2018). Consequently, the optogenetics applied to the PNS would never be useful unless they could be selectively applied to a specific group of neurons. If the DRG cell type‐specific optogenetics is established, it could manipulate each class of afferent neurons with sufficient precision, which would be applicable for not only medical treatment but also *in vivo* experiments where electrical stimulation has been used exclusively to activate the PNS (Devor & Wall, 1990; Waikar *et al*. 1996; Harris Bozer *et al*. 2019). For example, the use of this technique may allow the selective activation of Ib fibres without activating Ia fibres, which is difficult to achieve by electrical stimulation because the difference in the current required to recruit each type of fibres is subtle (Bradley & Eccles, 1953). Transgenic mice/rats engineered to express optogenetic tools in a cell‐specific manner offered a powerful approach to manipulate specific nerve fibres in the PNS (Ji *et al*. 2012; Liske *et al*. 2013), although the major disadvantage of this method is that it is largely limited to rodents and lower animals.

Recent studies have reported the use of adeno‐associated virus (AAV) vectors to deliver genes of interest into different DRG neurons because distinct AAV serotypes have different cellular tropisms (Mason *et al*. 2010). For example, AAV6 achieved high transduction levels of marker genes in DRG neurons with small‐diameter nociceptive afferents (Towne *et al*. 2009; Yu *et al*. 2013; Iyer *et al*. 2014, 2016). In contrast, AAV8 and AAV9 were very effective at delivering genes to large‐sized DRG neurons (Jacques *et al*. 2012; Schuster *et al*. 2014). AAV9 is widely used in gene therapy applications because it is considered harmless (Dayton *et al*. 2012; Murrey *et al*. 2014; Schuster *et al*. 2014; Hocquemiller *et al*. 2016), therefore this serotype might be suitable for targeting large‐sized DRG neurons with large‐diameter, non‐noxious afferents. To date, studies have reported the successful transduction of opsins into noxious afferents and demonstrated the selective manipulation of their activity (Boada *et al*. 2014; Iyer *et al*. 2014, 2016; Li *et al*. 2015); however, a comparable attempt has not been reported for their use in larger‐diameter, non‐noxious afferents.

The aim of this study was to manipulate the activity of DRG neurons with large‐diameter, cutaneous and proprioceptive afferents by means of optogenetics, and demonstrate that the optogenetic manipulation of PNS activity is feasible for the physiological study of synaptic transmission *in vivo*. To achieve this, we tested whether AAV9 preferentially transduces genes to large‐sized cells and compared its cellular tropism with AAV6, which has a preference for small‐sized cells. Subsequently, we transduced a light‐activated ion channel, Channelrhodopsin 2 (ChR2), into DRG neurons using AAV9. Then, upon confirmation of the successful delivery of ChR2 into the large‐sized DRG neurons, we tested whether their activity could be manipulated by light stimulation. We specifically compared the afferent volley induced by optical and electrical stimulation because the properties of action potentials induced by the two methods were reported to be different *in vitro* or *in silico* preparation (Williams & Entcheva, 2015; Ratnadurai‐Giridharan *et al*. 2017). Our results show that (1) AAV9 preferentially transduces medium‐to‐large‐sized DRG neurons, (2) compared with electrically elicited responses, the optically generated volleys in transduced DRG neurons have a higher sensitivity with amplitude of optical stimulation, but a lower sensitivity with stimulus frequency, and (3) afferent volleys evoked by light are sufficient to activate and recruit spinal reflex circuits. Overall, we report a method to manipulate the activity of cutaneous and proprioceptive afferents using optogenetics.

## Methods

### Ethical approval

All surgery and experimental protocols were carried out with approval from the local ethics committee for animal research at the National Institute of Neuroscience, Japan. The investigators understand the ethical principles under which *The Journal* operates and that their work complies with this animal ethics checklist.

### Experimental animals

The experiments were performed in 18 male Jcl:Wistar rats (4 weeks of age, body weight 60–100 g) obtained from CLEA Japan, Inc. (Tokyo, Japan). All animals were housed under a normal light/dark cycle (12 h:12 h) where light was on from 08.00 h until 20.00 h in a temperature‐controlled environment with food and water available *ad libitum*.

### Production of viral particles

AAV‐CMV‐AcGFP vectors serotype 6 and 9 (titres: 2.5 × 10^14^ genome copies ml^−1^) and AAV‐hSyn‐ChR2(H134R)‐EYFP vector serotype 9 (2.5 × 10^13^ genome copies ml^−1^) were produced using the helper‐free triple transfection procedure and purified by CsCl gradient or affinity chromatography (GE Healthcare, Piscataway, NJ, USA). Viral titres were determined by quantitative PCR using Taq‐Man technology (Life Technologies, Gaithersburg, MD, USA). The purity of vectors was assessed by 4%–12% sodium dodecyl sulfate‐acrylamide gel electrophoresis and fluorescent staining (Oriole, Bio‐Rad, Hercules, CA, USA). Transfer plasmids (pAAV‐CMV‐AcGFP‐WPRE and pAAV‐hSyn‐ChR2‐EYFP‐WPRE) were constructed by inserting the AcGFP‐WPRE sequence or hSyn promoter sequence and ChR2‐EYFP fragment (a kind gift from Dr Karl Deisseroth) with the WPRE sequence into an AAV backbone plasmid, respectively (pAAV‐CMV, Stratagene, La Jolla, CA, USA).

### Virus injection

**Figure 1:**
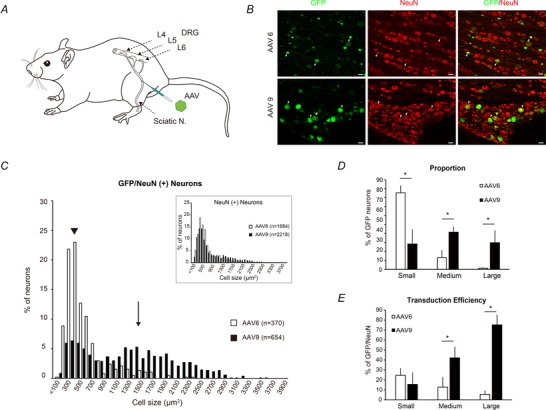
Cell‐size profile of GFP‐positive neurons transduced by AAV6 and AAA9 in dorsal root ganglia (DRG) *A*, schematic diagram of experiment. Gene transfer to DRG neurons was achieved by retrograde transport from the axon via sciatic nerve injection. *B*, DRG sections 4 weeks after AAV6‐GFP or AAV9‐GFP injection. Sections were immunostained with antibodies to GFP (green) and NeuN (red). Arrowheads indicate the same neuron co‐labelled with GFP and NeuN. Scale bars: 50 µm for all images. *C*, cell‐size frequency histogram for GFP/NeuN‐positive neurons in AAV6 (open bars) and AAV9 (filled bars) transduction groups. The histogram was generated in bins of 100 µm^2^. Arrowhead and arrow indicate the median size of GFP‐labelled neurons in AAV6 (arrowhead: 400 µm^2^) and AAV9 (arrow: 1400 µm^2^) transduction groups. Inset shows cell‐size frequency histogram for all neurons (NeuN positive). *D* and *E*, the proportion of GFP‐positive neurons (*D*) and transduction efficiency (*E*) within each cell‐size category (small: <700 µm^2^, medium: 700–1700 µm^2^, large: >1700 µm^2^) in AAV6 (open bars) and AAV9 (filled bars) transduction groups. AAV6 preferentially targets small‐sized DRG neurons. In contrast, AAV9 preferentially targets medium‐to‐large‐sized DRG neurons, showing high transduction efficiencies in large‐ and medium‐sized DRG neurons. ^*^Statistical difference (*P* < 0.05, Mann–Whitney *U* test) between AAV6 and AAV9‐labelled neurons.

Animals were anaesthetized by intraperitoneal injection of pentobarbital sodium (30 mg kg^−1^) and intramuscular injection of butorphanol (0.1 mg kg^−1^). Adequate anaesthesia depth was monitored frequently by checking the pupil size and flexion reflex to paw pinch. Mannitol solution (20%, 1 ml) was injected intraperitoneally 30 min before the virus injection to enhance the transduction efficiency of AAV vectors in DRG neurons (Vulchanova *et al*. 2010). We aimed to transfer genes to DRG neurons through retrograde transport by a sciatic nerve injection of AAV (Fig. 1 *A*). The rationale for this approach was that injection via the sciatic nerve was reported to achieve a higher transduction efficiency to the target DRG neurons on a segment basis compared with other methods (e.g. intravenous, intramuscular and intrathecal injections) (Towne *et al*. 2009; Vulchanova *et al*. 2010; Schuster *et al*. 2014), and is less invasive (Mason *et al*. 2010; Fischer *et al*. 2011; Iyer *et al*. 2016), both of which are advantageous for *in vivo* electrophysiological experiments. For sciatic nerve injection, an incision was made in the left thigh to expose the nerve. After isolating the sciatic nerve from surrounding tissue, a tapered glass capillary (200–300 µm tip, G‐1, Narishige Inc., Tokyo, Japan) was inserted into the sciatic nerve in the proximal thigh. The capillary was mounted on a micromanipulator and attached to a Hamilton syringe (1702RN, GL Science Inc., Tokyo, Japan) with polyethylene tubing (JT‐10, EICOM Inc., Kyoto, Japan). Tubing, syringe and capillary were filled with an electrically insulating stable fluorocarbon‐based fluid (Fluorinert, 3M, St Paul, MN, USA). We added 1% Fast Green (1 µl) to the viral vector solutions to visualize the injected solution. Before starting the injection, the capillary was left in place for 5 min to help seal tissues around the penetration point, and then 6 µl of viral vector solution was injected by a microinjection pump (NanoJet Quasi‐S, ISIS Co., Ltd, Seoul, South Korea) at 0.6 µl min^−1^. The capillary was removed 10 min after the injection to ensure absorption of the solution. The wound was closed with a non‐absorbable suture and animals were allowed to recover at 37°C.

### Experiment 1: cellular tropism of AAV6 and AAV9

We used AAV‐CMV‐AcGFP vector serotypes 6 and 9 (titres: 2.5 × 10^14^ genome copies ml^−1^) to achieve gene transfer into DRG neurons. A viral vector of each serotype was injected into the left sciatic nerve of four animals per serotype. Four weeks after injection, all rats were killed and perfused for later histological analysis. Briefly, animals were deeply anaesthetized by intraperitoneal injection of pentobarbital sodium (50 mg kg^−1^) and transcardially perfused with phosphate‐buffered saline (PBS; pH 7.4), followed by 300 ml of 4% paraformaldehyde (PFA). Thereafter, the lumbar region of the spinal cord, together with the DRG and sciatic nerve, were sampled, post‐fixed in 4% PFA overnight at 4°C, and transferred to 30% sucrose in PBS at 4°C.

#### Immunohistochemistry

Frozen L4 and L5 DRGs were sectioned at 20 µm thickness on a cryostat and mounted onto gelatinized slides. After washing three times with PBS, the sections were incubated with PBS containing 2% normal goat serum (NGS) for 1 h at room temperature, followed by incubation with primary antibody diluted in 2% NGS and 0.1% Triton X‐100 in PBS overnight at 4°C. Then, the sections were washed with PBS three times and incubated with secondary antibody diluted in 2% NGS in PBS for 1 h at room temperature. Sections were washed with PBS and covered by a glass coverslip. Control sections were stained using the same protocol but the primary antibodies were omitted. All processes were performed in a dark chamber. The primary antibodies were as follows: chicken anti‐GFP (Abcam, Cambridge, UK) at 1:1000 and rabbit anti‐NeuN (Abcam) at 1:2000. The secondary antibodies were as follows: goat anti‐chicken IgG (Abcam, Alexa488) at 1:500 and donkey anti‐rabbit IgG (Abcam, Alexa555) at 1:500.

#### Histological quantification

The distribution of cell size for green fluorescent protein (GFP)‐positive neurons was measured to determine the difference in the cellular tropism of AAV6 and AAV9 for DRG neurons. To quantify the cell‐size profiles, only cells with a visible nucleus stained by NeuN antibody were counted. GFP‐positive neurons were defined as cells with a fluorescence intensity greater than the average background fluorescence plus two standard deviations in a section from a naive tissue with no viral vector injection. Every 10th DRG section spaced by 200 µm was selected from consecutive serial sections and four to six sections were obtained per rat (2–3 sections from each DRG). The section was photographed at fixed exposure settings at ×10 magnification using an inverted fluorescence microscope (BZ‐X710; Keyence, Japan). Image analysis and quantification were performed using image analysis software for the BZ‐X710 (BZ‐H3XF). In each selected section, the number of GFP‐labelled cells was counted. The cross‐sectional area of each GFP‐labelled cell was measured to construct a histogram of cell‐size distribution. Total DRG neuronal populations were identified by using the neuron marker NeuN. In NeuN‐positive neurons, the same processes were performed as for GFP‐positive neurons.

### Experiment 2: optogenetic activation of DRG neurons

**Figure 2:**
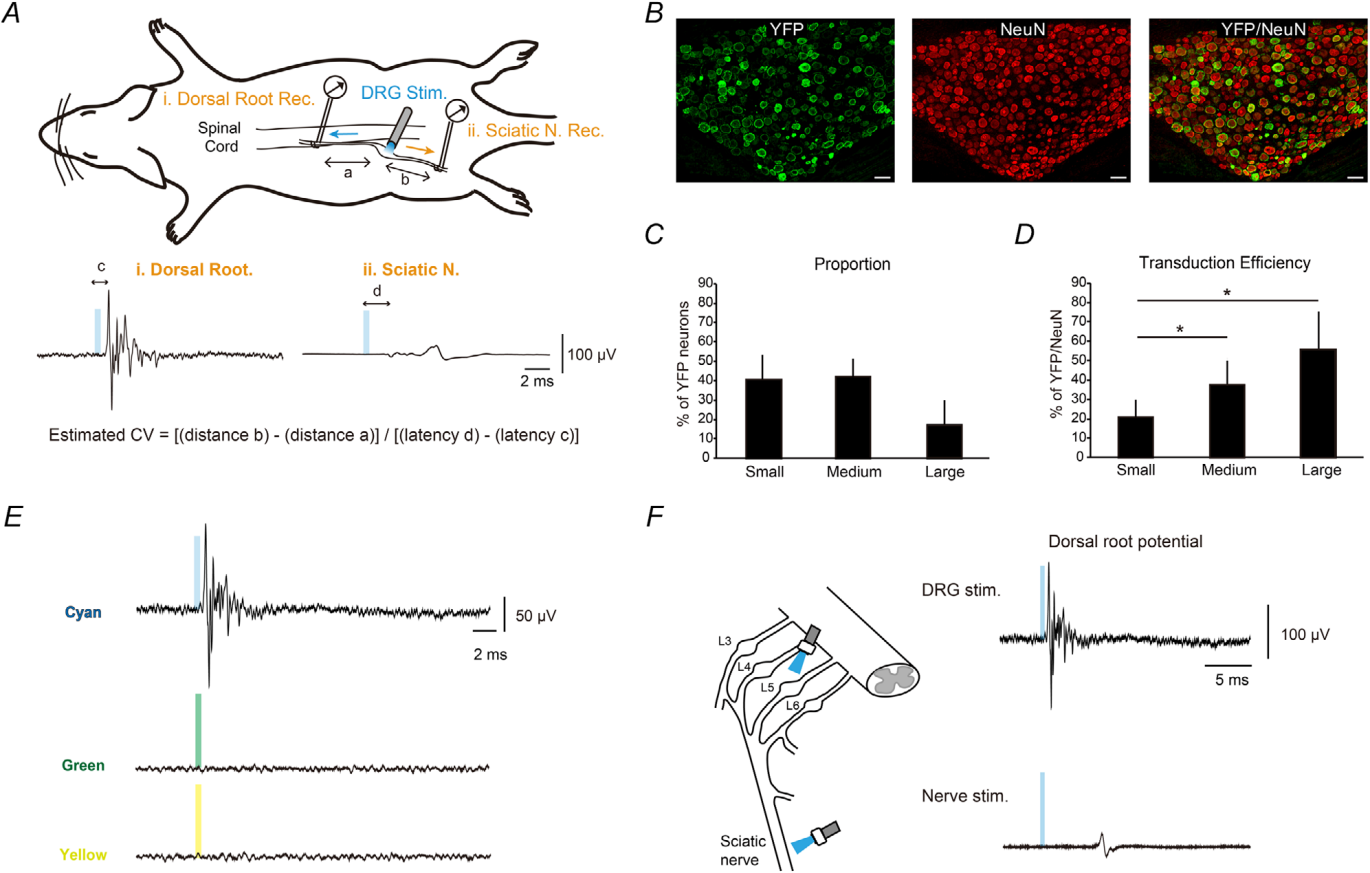
Optogenetic activation of fast‐conducting, primary afferent fibres by AAV9‐mediated ChR2 transduction in DRG neurons *A*, schematic diagram of experiment. L4–5 DRG neurons were illuminated with blue light and orthodromic/antidromic volleys were recorded from the dorsal root (i) and the sciatic nerve (ii). In the electrical stimulation experiment, the sciatic nerve was stimulated and the response was compared with that induced by light stimulation. *B*, representative YFP‐expressing DRG cell images. Sections were stained with GFP (green) and NeuN (red) antibodies. Scale bars: 100 µm for all images. *C* and *D*, the proportion of YFP‐positive neurons (*C*) and transduction efficiency (*D*) among each cell‐size category (*n* = 10). AAV9 with an hSyn promoter targeted medium‐to‐large‐sized DRG neurons, showing high transduction efficiencies in large‐ and medium‐sized cells. ^*^Statistical difference (*P* < 0.05, Tukey HSD test) between cell‐size categories. *E*, example of light stimulation‐induced volleys recorded from the dorsal root under different wavelengths of LED light. Timing of the applied light pulse is represented by coloured bars. Each waveform shows the mean value of 20 responses. *F*, example of light stimulation‐induced volleys recorded from the dorsal root. Light stimulation was applied to the DRG (upper) or the sciatic nerve (lower) at a fixed intensity (58.4 mW, pulse width: 500 µs). Volleys at the dorsal root were elicited by both stimulation types, but the responses evoked by nerve stimulation were smaller than that evoked by DRG stimulation.

A previous study reported that AAV harbouring a cytomegalovirus (CMV) promoter might be toxic for neurons, whereas neuron‐specific Ca^2+^–calmodulin‐dependent protein kinase II (CaMKII) or synapsin I (synI) promoters were not toxic (Watakabe *et al*. 2015). Therefore, for the optogenetic experiment, we used an human synI (hSyn) promoter and transduced ChR2 into DRG neurons by AAV9 (AAV‐hSyn‐ChR2‐enhanced yellow fluorescent protein (EYFP) vector serotype 9, 2.5 × 10^13^ genome copies ml^−1^) via sciatic nerve injection as for experiment 1. After 3–6 weeks, a terminal experiment was performed. Animals were anaesthetized by an intraperitoneal injection of pentobarbital sodium (40 mg kg^−1^) and intramuscular injection of butorphanol (0.1 mg kg^−1^). Adequate anaesthesia depth was monitored frequently by checking the pupil size and flexion reflex to paw pinch, and maintained with additional pentobarbital sodium (10 mg kg^−1^, intravenous) and butorphanol (0.1 mg kg^−1^, intramuscular) administration. During the experiment, rats were tracheostomized and intubated with a cuff tube, and then ventilated with a respirator (SN‐480‐7, Shinano Manufacturing, Tokyo, Japan) at a tidal volume of 10 ml kg^−1^. Next, an external jugular vein was cannulated to administer fluid and drugs. Rat body temperature was maintained within a physiological range. The lumbar spinal cord was exposed by performing a laminectomy from the 12th thoracic vertebra to the 6th lumbar vertebra, and the upper thoracic spine and tail were immobilized in a metal frame using clamps. The dura was gently opened with a sharp needle, and L4/L5 dorsal and ventral roots were isolated from other roots. An Ag–AgCl bipolar hook electrode (fabricated by our laboratory) was mounted on the isolated dorsal root at 15–25 mm proximal to the DRG to record evoked potentials. The left sciatic nerve was exposed and isolated from the surrounding tissues, and the other Ag–AgCl bipolar hook electrode was mounted on the isolated sciatic nerve to record the antidromic volley or to apply the electrical stimulation (Fig. 2 *A*). The skin and muscle around the exposed tissues were raised and tied to form a pool, which was filled with mineral oil to protect the exposed cord or nerve. After surgical operation, rocuronium bromide (0.1 mg kg^−1^, intravenous) was administered to achieve neuromuscular blockade.

The L4–L5 DRGs were illuminated with blue light (spectra: 470 nm) using an optical stimulation device (Spectra X light engine, Lumencor Inc., Beaverton, OR, USA) and evoked responses were recorded at the dorsal root and the sciatic nerve (Fig. 2 *A*). An optic probe of 1 mm diameter was positioned perpendicular to the axis of the DRG so that the diameter of the light spot was roughly equivalent to the diameter of the DRG. The response properties of DRG neurons to light stimulation were confirmed under various conditions. First, the effects of pulse duration on the evoked responses were examined by changing the pulse width from 0.1 to 10 ms at a fixed intensity (58.4 mW). Second, intensity–response characteristics of the evoked responses were examined by changing the stimulation intensity from 2.16 to 78.0 mW at a fixed pulse width (500 µs). Third, to examine response sensitivity, a paired‐pulse stimulation (pulse width: 500 µs, intensity: 58.4 mW) was applied repeatedly. The interval between two pulses was changed from 5 to 20 ms in 2.5 ms steps. Furthermore, the evoked responses induced by light were compared with those induced by electrical stimulation in the same animals. For electrical activation of the primary afferent and DRG neurons, the left sciatic nerve was stimulated using a constant current isolator (SS 102L, Nihon Kohden, Tokyo, Japan) coupled with an electrical stimulator (SEN7203, Nihon Kohden). Intensity–response characteristics were examined by changing the stimulation intensity (pulse width: 100 µs, intensity: 0.5–10 times the threshold, 20–1000 µA) and the response sensitivity was examined by a paired‐pulse stimulation protocol (pulse width: 100 µs, intensity: twice the threshold, 40–100 µA). The L4–L5 DRG were also illuminated with green (spectra; 550 nm) or yellow (spectra; 575 nm) light at a fixed intensity (58.4 mW, pulse width: 500 µs) to test whether light stimulation effects were caused by the activation of ChR2. Finally, to test whether the dorsal root potentials evoked by light recruited postsynaptic neurons in the spinal reflex arc, ventral root potentials were simultaneously recorded in two rats. The stimulation parameters were fixed as follows: pulse width: 500 µs, intensity: 58.4 mW. After confirming ventral root potential, the L4/L5 dorsal roots were transected, and responses of the ventral root were checked again to eliminate potential direct stimulation effects of motor axons. The responses recorded from the dorsal or ventral roots were averaged over 20 trials for each condition and the stimulation pulse was delivered with an interval of 1 s. The volley signals were amplified (×1000) and band‐pass filtered (50 Hz–10 kHz), using an amplifier (MEG‐6108M, Nihon Kohden). The signals were digitized using an analog/digital converter with a sampling rate of 50 kHz (Digidata 1550, Molecular Devices, LLC, San Jose, CA, USA), and stored on a hard disk for subsequent analysis. The recording period was 100 ms including a pre‐stimulus period of 10 ms. The evoked responses were measured as peak‐to‐peak amplitudes of the non‐rectified volley and the conduction time between the onset of stimulus and the beginning of the response was measured as the latency. The beginning of the response was defined as a point that exceeded the value of the mean ± three standard deviations of the background signals recorded 10 ms before the stimulation. To estimate the conduction velocity under optical stimulation conditions, we calculated the conduction velocity by dividing the subtracting distance between the DRG and dorsal root (Fig. 2 *A*; distance a) and the sciatic nerve (distance b) by the difference in latency of the orthodromic volley (latency c) and antidromic volley (latency d). Under electrical stimulation conditions, after subtracting an estimated stimulus utilization time of 0.1 ms (McDonald, 1963) from the conduction time, the conduction velocity was calculated by dividing the conduction time by the conduction distance. The utilization time of optical stimulation to DRG neurons was obtained as follows: (1) the arithmetic conduction time for the distance between the DRG optical probe to the recording electrode at the sciatic nerve was calculated by means of the conduction velocity of optically elicited volleys (see above); (2) we used the actual onset latency from DRG optical stimulation to the volley at the sciatic nerve; and (3) we obtained the utilization time by subtracting (2) from (1). Note that the utilization time is strongly affected by the distance between the recording point and stimulating point at a shorter interelectrode distance (Ruscheweyh *et al*. 2007). Here, we used a volley recorded at the sciatic nerve for the calculation.

At the end of the experiments, small electrolytic lesions were created in the dorsal root and sciatic nerve by passing a 40 µA direct current through the recording and stimulating bipolar hook electrode for 40 s. The animals were deeply anaesthetized by an intravenous injection of pentobarbital sodium (20 mg kg^−1^) and then transcardially perfused with PBS (pH 7.4), followed by 300 ml of 4% PFA. The lumbar region of the spinal cords together with the DRG and sciatic nerve were removed, and then the distance between the mounting site of the bipolar hook electrode in the sciatic nerve and DRG, and between the dorsal root recording site and DRG, were measured. To quantify yellow fluorescent protein (YFP) expression in DRG neurons, DRG sections were stained with antibodies against GFP, as previously described (Iyer *et al*. 2016).

### Data analysis

A two‐sample Kolmogorov–Smirnov test was used to compare differences in the distributions of cell‐size frequency between AAV6 and AAV9. In experiment 1, the proportion and transduction efficiency of GFP‐positive neurons within each cell size category was compared using the Mann–Whitney *U* test. In experiment 2, the transduction efficiency of GFP‐positive neurons for each cell‐size category was compared by one‐way analysis of variance (ANOVA) and the Tukey HSD test was used for multiple comparisons. The estimated conduction velocity of volleys evoked by light or electrical stimulation was compared using the Wilcoxon signed rank test. In a paired‐pulse stimulation protocol, the sensitivity of the second response was compared with the first response, using a one sample *t* test. The data values are presented as the means ± standard deviation. *P* values <0.05 were considered significant in all statistical analyses. SPSS version 22 software (IBM SPSS, IBM Japan Ltd, Tokyo, Japan) was used for Mann–Whitney *U* tests, one‐way ANOVA, the Tukey HSD test, Wilcoxon signed rank test and one sample *t* tests. Matlab_R2014b statistical toolbox (MathWorks, Tokyo, Japan) was used for two‐sample Kolmogorov–Smirnov tests.

## Results

### Cell‐size profile of GFP‐positive neurons transduced by AAV6 and AAV9 vectors

In this experiment, eight male rats were used to determine the cellular tropism of AAV6 and AAV9 vectors expressing GFP for DRG neurons. We observed GFP expression in the L4 and L5 DRG neurons following the injection of these AAV vectors into the left sciatic nerve. GFP‐expressing cells were co‐labelled with NeuN, a neuron marker, suggesting that GFP was predominantly expressed in cell bodies and axons of neurons (Fig. 1 *B*). Then, we counted the number of cells simultaneously stained with both markers. Quantitative analysis showed that the mean percentage of GFP‐positive neurons was 21.24 ± 7.0% for AAV6 and 30.03 ± 9.13% for AAV9. The cell‐size distribution of GFP‐positive neurons, measured as total area of their cell body, was significantly different between AAV6 and AAV9 (two‐sample Kolmogorov–Smirnov test, *P* = 0.0024, Fig. 1 *C*), while the distribution of NeuN‐positive neurons was similar for both serotypes (*P* = 0.1628, Fig. 1 *C*; inset). The distribution histograms showed that GFP‐positive neurons transduced by AAV6 were preferentially distributed to the left side (arrowhead: median cell size = 400 µm^2^). In contrast, neurons transduced by AAV9 were widely distributed to the right side (arrow: median cell size = 1400 µm^2^). After AAV6 injection, DRG neurons were categorized on the basis of size into small (<700 µm^2^), medium (700–1700 µm^2^) and large (>1700 µm^2^) cells as described previously (Harper & Lawson, 1985; Hammond *et al*. 2004; Jamieson *et al*. 2005), and 85.48 ± 7.9% of GFP‐positive neurons were classified as small‐sized cells, 13.26 ± 8.17% as medium‐sized and 1.25 ± 1.02% as large‐sized (Fig. 1 *D*). After AAV9 injection, 41.57 ± 6.27% of GFP‐positive neurons were classified as medium‐sized cells, 29.89 ± 13.37% as large‐sized cells and 28.53 ± 16.37% as small‐sized cells. The proportion of GFP‐positive neurons transduced by each serotype was significantly different between each cell‐size category (Mann–Whitney *U* test, *P* = 0.021). Moreover, the transduction efficiency within each cell‐size category showed an apparent difference between AAV6 and AAV9 (Fig. 1 *E*). AAV9 showed a high proportion of GFP‐positive neurons within large‐sized cells (75.12 ± 10.34%) compared with a lower proportion by AAV6 (5.53 ± 3.75%; Mann–Whitney *U* test, *P* = 0.021). The transduction efficiency of the AAV9 vector was moderate for medium‐sized cells (42.05 ± 11.10%) but still significantly higher than for AAV6 (12.79 ± 9.95%) (Mann–Whitney *U* test, *P* = 0.021). However, AAV9 had a lower transduction efficiency for small‐sized cells (15.60 ± 12.01%) compared with AAV6 (24.48 ± 7.34%), although this was not statistically significant (Mann–Whitney *U* test, *P* = 0.356). It is well established that medium‐to‐large‐sized DRG neurons have thickly myelinated Aα/β‐fibres, which transmit mechanoreceptive and proprioceptive signals to the CNS, and that small‐sized DRG neurons have unmyelinated C‐fibres and thinly myelinated Aδ‐fibres, which transmit nociceptive signals (Harper & Lawson, 1985; Honda, 1995; Hammond *et al*. 2004; Jamieson *et al*. 2005; Li *et al*. 2016). Therefore, AAV9 preferentially transduced mechanoreceptive and proprioceptive afferents with a gene encoding GFP. Next, we transduced ChR2 into DRG neurons via AAV9 and tested the electrophysiological properties of transduced DRG neurons.

### Comparison of electrically and optically elicited dorsal root potentials

We transduced ChR2 into the DRG neurons of 10 rats by AAV9 via sciatic nerve injection, and attempted to activate ChR2‐expressing DRG neurons by applying blue light. A representative result in one rat is shown in Fig. 2 *B*, *E* and *F*. After an adequate survival period (3–6 weeks), YFP expression, an indication of the successful transduction of ChR2, was observed in the L4 and L5 DRG neurons (Fig. 2 *B*), and the proportion (Fig. 2 *C*) and transduction efficiency (Fig. 2 *D*) of these YFP‐positive neurons between each cell‐size category were similar to those for CMV (Fig. 1 *D* and *E*); the transduction efficiency to medium‐ and large‐sized cells was significantly higher than to small‐sized cells (*n* = 10, Tukey HSD test: *vs*. medium, *P* = 0.044; *vs*. large, *P* = 0.001). Moreover, when DRG neurons were stimulated by blue light, compound action potentials (CAPs) were recorded at their dorsal root, while green or yellow light stimulation of the DRG neurons did not induce any responses (Fig. 2 *E*). These results were confirmed in all animals tested (*n* = 10). In addition, we also observed dorsal root potentials when the light stimulation was applied to the sciatic nerve (pulse width: 500 µs, intensity: 58.4 mW) (Fig. 2 *F*). However, responses by optical nerve stimulation were less stable and smaller compared with DRG stimulation, probably because the physical instability of peripheral nerves makes it difficult to fix the probe on the nerve, and because of the inefficiency of activating sensory afferents by optical stimulation to mixed sciatic nerves compared with DRG cells consisting of pure sensory neurons. Therefore, we examined dorsal root potentials evoked by the light stimulation of DRG neurons, and compared them with electrically elicited responses.

**Figure 3:**
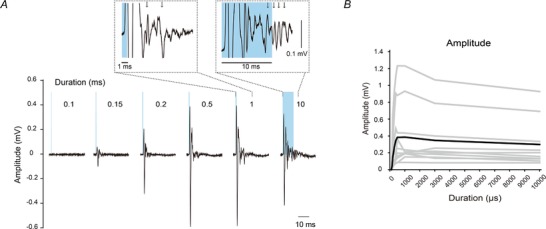
Response properties of Channelrhodopsin 2‐expressing DRG neurons at varying light pulse durations *A*, example of light stimulation‐induced volleys recorded from the dorsal root under different pulse durations. Timing of the applied light pulse is represented by blue bars. Each waveform shows the mean value of 20 responses. Insets show an enlarged view of late component volleys from stimulation onset to the end of volleys (scale bar: 0.1 mV). Arrows indicate individual peaks of late component volleys. *B*, amplitude of evoked compound action potential as a function of pulse duration. Grey lines represent individual rats and the black line indicates the mean value of all rats (*n* = 10). The peak amplitude was rapidly increased up to 1 ms and then slightly decreased according to the increment of pulse duration.

**Figure 4:**
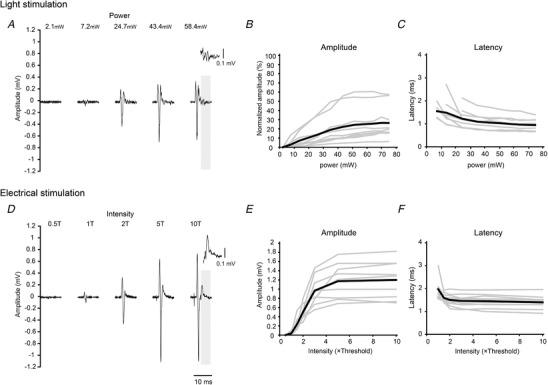
Recruitment characteristics of Channelrhodopsin 2‐expressing DRG neurons as a function of stimulation intensity *A*, example of light stimulation‐induced volley recorded from the dorsal root under different stimulus intensities. Shadow area represents the estimated range of Aδ‐fibre responses and the inset shows an enlarged view of this time range (scale bar: 0.1 mV). Each waveform shows the mean value of 20 responses. *B*, amplitude of light‐evoked compound action potential (CAP) as a function of light power. Data were normalized by the maximal amplitude of the electrically evoked CAP. *C*, conduction time of the evoked CAP relative to the onset of light stimulation as a function of light power. Grey lines in *B* and *C* represent individual rats and black lines indicate the mean value of all rats (*n* = 9). *D–F*, example of volleys elicited by electrical stimulation with different stimulus intensities. Same figure format as in *A–C*. T: threshold. Note that the recruitment characteristics are comparable between light and electrically elicited responses.

**Figure 5:**
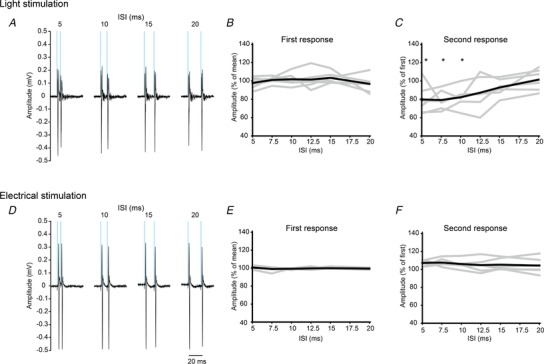
Response sensitivity to dual stimuli with different intervals *A*, examples of light stimulation‐induced dorsal root volleys under different interstimulus intervals (ISIs). Timing of the stimulation pulse is represented by blue lines. Each waveform shows the mean value of 20 responses. *B* and *C*, amplitude of optically elicited CAP by first stimulation (*B*) and second stimulation (*C*) as a function of ISI. Data were normalized by the mean amplitude of the first response in *B* and *C*. Grey lines represent individual rats and black lines indicate the mean value of all rats (*n* = 6). *D–F*, examples of electrical stimulation‐induced dorsal root volleys under different ISIs. Same figure format as for *A–C*. ^*^Statistical difference (*P* < 0.05, one‐sample *t* test) compared with baseline control.

**Figure 6:**
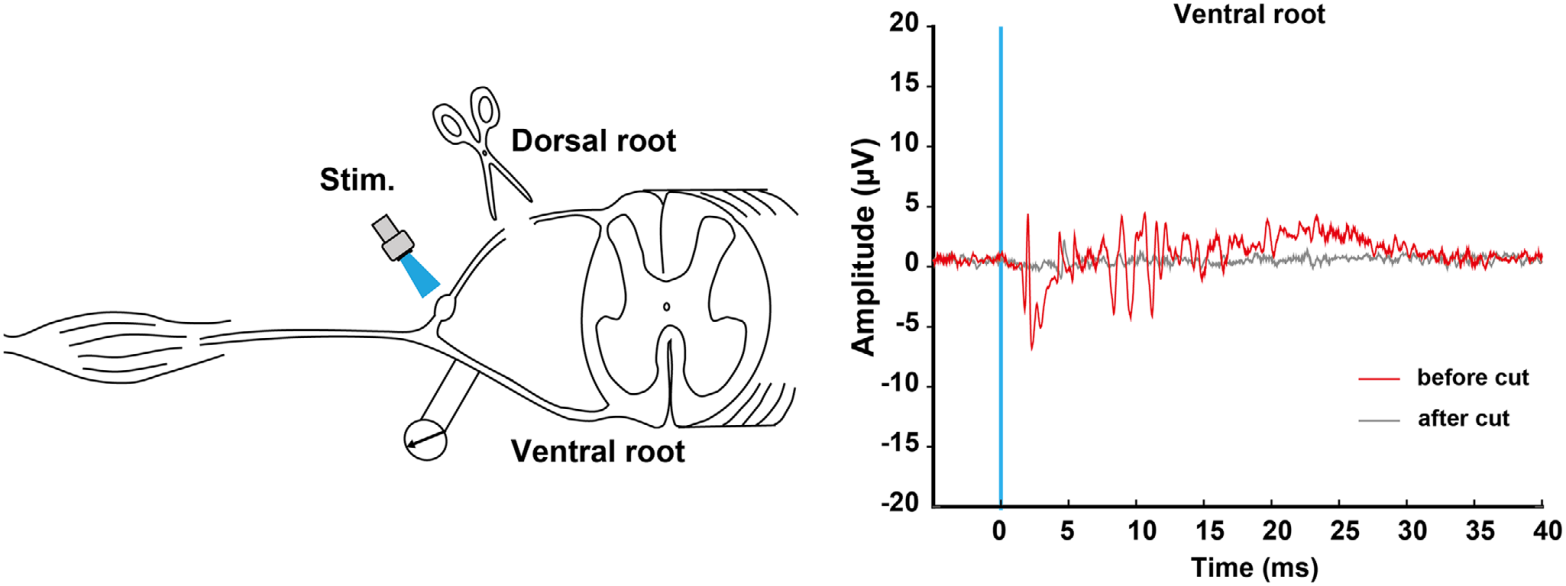
Optogenetic recruitment of spinal reflex Ventral root potential evoked by light stimulation was recorded before (red) and after (grey) dorsal root cut. Each waveform shows the mean value of 20 responses. Timing of light pulse is represented by the blue line. Light stimulation activated spinal motor neurons. Representative data from one rat.

We found unique characteristics for the optically elicited volleys as shown in Figs 3, 4, 5, 6. First, the evoked response varied depending on the pulse duration. The peak amplitude was rapidly increased according to the increment of pulse duration and it was saturated around 1 ms (Fig. 3 *A*). The evoked responses showed multiple peaks with different sizes for a longer pulse duration (Fig. 3 *A*; inset), suggesting that multiple cells with different conduction velocities as well as different utilization times were activated to form the CAPs, although the peak with maximal magnitude was always found to be the first peak. Because these characteristics were observed in all rats (Fig. 3 *B*), the use of short pulse durations (around 500 µs) appeared to be better than longer pulse durations to selectively elicit the early components of CAPs.

Second, the amplitude of evoked responses increased with increasing stimulation intensity until it reached a maximum, and the onset latency of the responses was shortened associated with the increased amplitude of responses (Fig. 4 *A–C*). The maximum amplitude of optically elicited volleys (mean; 0.36 ± 0.3 mV) was smaller than that induced by electrical stimulation by approximately 30% (mean; 1.17 ± 0.39 mV), although there was some variation in the responses (Fig. 4 *B* and *E*). Moreover, the late components of CAPs were only observed at higher stimulation intensities (Fig. 4 *A*; grey shadow area). These intensity–response characteristics are similar to those of electrical stimulation (Fig. 4 *D–F*).

Third, conduction velocity and utilization time of optically and electrically elicited responses were different. The conduction velocity was calculated from seven (out of 10) rats where we simultaneously recorded the orthodromic/antidromic volleys. The estimated conduction velocity of optically elicited volleys ranged from 11.57 to 37.58 m s^−1^ (mean; 23.84 ± 9.95 m s^−1^), while that induced by electrical stimulation ranged from 43.42 to 93.90 m s^−1^ (mean; 61.12 ± 15.84 m s^−1^). The conduction velocity was significantly different between the two stimulation conditions (Wilcoxon signed rank test, *P* = 0.018). The utilization time for optical stimulation was estimated as 0.3 ± 0.51 ms, which was also longer than that induced by electrical stimulation (0.1 ms; McDonald, 1963). Differences in the conduction velocity were ascribed to the intrinsic mechanism for generating action potentials by stimulation methods. While recruitment of afferent fibres by electrical stimulation strictly follows electrophysiological properties (i.e. recruitment occurs from fast‐conducting fibres; Lertmanorat & Durand, 2004; Llewellyn *et al*. 2010), DRG neurons with the best ChR2 transduction efficiency that should elicit the earliest and largest volley may not always be the fastest conducting neurons. A previous study reported that the recruitment order of afferent fibres by optical stimulation followed the expression levels of ChR2 (Srinivasan *et al*. 2018). Therefore, we reasonably expected a slower conduction time in the optically elicited volley. A longer utilization time might be related to the heterogenicity of transduction efficiency among medium‐to‐large‐sized DRG neurons. Upon light stimulation, voltage‐gated sodium channels were activated by changes in the electrical membrane potential through light‐gated cation channels (Zhang *et al*. 2011; Rost *et al*. 2017), which would result in various utilization times to generate action potentials depending on the ChR2 expression levels. Therefore, a longer utilization time is also expected. Nevertheless, most of the estimated conduction velocities observed under optical stimulation conditions were within the Aα/β‐fibre range (>14 m s^−1^), not the Aδ‐fibre (<8 m s^−1^) or C‐fibre (<1.4 m s^−1^) range, as estimated using animals of the same age and strain (Harper & Lawson, 1985). These recruitment characteristics may indicate that fast‐conducting, low‐threshold Aα/β fibres are mainly recruited by light stimulation, reflecting the high transduction efficiency within the medium‐to‐large‐sized DRG neurons (Fig. 2 *D*).

Fourth, the response sensitivity of repetitive stimuli was different between light and electrical stimulation (Fig. 5). Within short interstimulus intervals (ISIs) of 5, 7.5 or 10 ms, the amplitude of the second response was suppressed only by light stimulation (one sample *t* test, *P* = 0.031, 0.005, 0.012, respectively, Fig. 5 *C* and *F*), although the amplitude of the first response was unchanged in both conditions (Fig. 5 *B* and *E*). The decline in the amplitude of the second response indicated that ChR2 was desensitized under repetitive light stimulation (Hegemann & Moglich, 2011; Lin, 2011).

In summary, these analyses demonstrated that the optogenetic activation of fast‐conducting, putative Aα/β‐fibres was obtained through the use of a recombinant AAV9 vector, and that responses that follow ChR2 kinetics can be modulated by changing the pulse duration and intensity of the light stimulation.

### Optogenetic recruitment of spinal reflex pathways

Finally, to confirm whether the dorsal root potentials evoked by light were sufficiently effective to recruit postsynaptic neurons in the spinal reflex arc, the ventral root potential was recorded before and after a dorsal root cut (Fig. 6). A ventral root potential that was concomitant with dorsal root volleys disappeared following a dorsal root cut. Moreover, the latency of the evoked responses was shorter than that of the nociceptive withdrawal reflex (Schouenborg & Sjolund, 1983). The same results were observed in another rat (data not shown). Our observation that optically elicited dorsal root volleys were sufficient to produce motor neuron depolarization, with the short onset latency of ventral root responses, may account for their preferential activation of cutaneous and proprioceptive afferents.

## Discussion

In this study, we found that AAV9‐mediated gene transduction in DRG neurons had a preference for medium‐to‐large‐sized cells, and that the optical stimulation of ChR2‐introduced DRG neurons produced CAPs originating from fast‐conducting nerve fibres. Moreover, we showed that afferent volleys evoked by light were sufficient to activate postsynaptic neurons in the spinal reflex arc. These results may yield new insights into the role of sensory afferent inputs to the CNS for behavioural control.

### AAV9 vector has cellular tropism for DRG neurons with medium‐to‐large‐diameter afferents

Earlier studies showed that individual AAV vector serotypes have different tissue tropisms (Zincarelli *et al*. 2008; Srivastava, 2016), which allows gene transfer to DRG neurons in a cell type‐specific manner (Mason *et al*. 2010). AAV6 was reported to be superior for gene transduction into small‐sized DRG neurons, which were mainly classified as primary nociceptive neurons consisting of unmyelinated C‐fibres and thinly myelinated Aδ‐fibres based on their morphological characteristics (Towne *et al*. 2009; Yu *et al*. 2013; Iyer *et al*. 2014, 2016). Our findings regarding the skewed distribution of GFP‐positive neurons to small‐sized cells by AAV6 vector transduction (Fig. 1 *C*) are consistent with these previous reports, indicating that this serotype is preferable for the optogenetic manipulation of pain‐related fibres.

In contrast, we found that approximately 70% of GFP‐positive neurons were categorized as medium‐to‐large‐sized cells after the sciatic nerve injection of an AAV9 vector (Fig. 1 *D*). Of these cells, large‐sized DRG neurons showed a high transduction efficiency (>75% were GFP positive), in contrast to the lower transduction efficiency of AAV9 for small‐sized cells (<16% were GFP positive) (Fig. 1 *E*). The cell‐size profiles of GFP‐positive neurons observed in this study support the notion that AAV9 preferentially targets medium‐to‐large‐sized DRG neurons transmitting mechanoreceptive and proprioceptive signals to the CNS (Harper & Lawson, 1985; Honda, 1995; Hammond *et al*. 2004; Jamieson *et al*. 2005; Li *et al*. 2016).

AAV9‐mediated gene transfer to DRG neurons has been confirmed in rodents as well as in non‐human primates (Gray *et al*. 2013). Moreover, the AAV9 vector was identified as an optimal gene transduction carrier for gene therapy because this serotype is presumed to not induce toxicity or tissue damage (Murrey *et al*. 2014; Schuster *et al*. 2014). Therefore, it is plausible that selective ChR2 transduction into large‐sized DRG neurons, as demonstrated in this paper, might also be applicable to non‐human primates or human patients. In future studies, a comparison of the cell‐type specificity between rodents and primates should be performed to apply our findings to non‐human primates.

### Intra‐nerve injection is an optimal route for the gene transduction of DRG neurons

Gene transfer to DRG neurons has been achieved by a number of delivery routes including subcutaneous, intramuscular, intravenous, intrathecal, intraganglionic and intra‐nerve administration (Towne *et al*. 2009; Vulchanova *et al*. 2010; Jacques *et al*. 2012; Yu *et al*. 2013; Schuster *et al*. 2014). This indicates that the tropism of AAV serotypes is unchanged irrespective of the injection method. However, these methods have some advantages and disadvantages in terms of their transduction efficiency and target selectivity. For example, the intraganglionic injection of an AAV vector enabled efficient gene expression on a segment basis with no obvious off‐target effects (Glatzel *et al*. 2000). However, from a methodological point of view, direct DRG injection is a highly invasive procedure that risks damaging DRG neurons. Because the DRG is covered by an intervertebral foramen, a partial laminectomy and large incision is required to access the DRG (Puljak *et al*. 2009; Fischer *et al*. 2011). Indeed, our preliminary experiments showed obvious cell damage and motor deficits after direct DRG injection (data not shown).

Subcutaneous, intravenous, intramuscular and intrathecal injections are less invasive methods. Previous studies suggested that intrathecal gene delivery to DRG neurons led to higher levels of gene expression; in contrast, other delivery routes showed a lower transduction efficiency (Towne *et al*. 2009; Schuster *et al*. 2014). However, the disadvantages of intrathecal injection include the lack of selectivity to specific segments and off‐target effects such as the unexpected transduction of liver cells (Towne *et al*. 2009; Schuster *et al*. 2014). The present study demonstrated that intra‐nerve injection produced efficient gene transfer to DRG neurons without damaging associated tissues; therefore, we propose that intra‐nerve injection is the optimal method for the transduction of DRG neurons (Fig. 1 *B*).

### Difference between electrically and optically induced afferent responses

We demonstrated the optogenetic activation of fast‐conducting, primary afferent fibres by AAV9‐mediated ChR2 transduction in DRG neurons. Although optically and electrically elicited volleys were similar in the primary sensory afferents (e.g. recruitment property; Fig. 4), there were specific characteristics related to responses induced by light stimulation.

First, unlike electrical stimulation, the response sensitivity for light stimulation was suppressed within a short ISI (<10 ms). This desensitization might be attributed to the temporal dynamics of neuronal activity, which is determined by the ChR2 conducting state (four‐state photocycle kinetics; two open and two closed) (Hegemann & Moglich, 2011; Srinivasan *et al*. 2018). When the second stimulation was applied before closing the light‐gated channels (Lin, 2011), some neurons may be shifted to the low‐conductance open state, leading to a low probability of neuronal depolarization. This lower sensitivity to high‐frequency optical stimulation should be taken into account when using high‐frequency, repetitive stimulation to magnify the effect by facilitating temporal summation in postsynaptic neurons.

Second, optical stimulation to recruit afferent fibres is restricted to those expressing ChR2, and the recruitment order was shown to follow the expression levels of ChR2 (Srinivasan *et al*. 2018). Therefore, our observation that AAV9‐mediated ChR2 expression was biased to large‐sized cells suggests that light stimulation preferentially recruits fast‐conducting afferents at a specific stimulation intensity. This is clearly different from the recruitment pattern of afferent fibres by electrical stimulation, which are recruited in order of fibre diameter; recruitment occurs from afferent fibres with a large diameter because they are more easily depolarized due to lower input resistance for a given applied voltage (Lertmanorat & Durand, 2004; Llewellyn *et al*. 2010). However, we observed that the recruitment of high‐threshold, late components, even by optical stimulation, had a strong intensity (Fig. 4 *C*). This probably represents the difficulty in expressing a gene exclusively in a targeted class of fibres using AAV vectors (Vulchanova *et al*. 2010; Yu *et al*. 2013; Iyer *et al*. 2014). Therefore, non‐target effects should be taken into account when applying light stimuli with a strong intensity.

### Potential application to basic and clinical research

Throughout this century, the fundamental structure and mode of action of the spinal reflex circuit have been determined by confirming the input–output relationship using the electrical stimulation of afferent fibres (Baldissera *et al*. 1981). The proven disadvantage of this approach is the low spatial resolution. Efficacy of electrical stimulation to activate afferent fibres is affected largely by the passive electrophysiological property of the applied electrical current, e.g. recruitment order (Lertmanorat & Durand, 2004; Llewellyn *et al*. 2010) or cable theory (Hodgkin & Rushton, 1946), and this property is frequently disadvantageous for the objective of physiological experiments. For example, afferent fibres from the Golgi tendon organ (Ib) or secondary ending of the muscle spindle (II) possess a slightly smaller diameter than those with a primary ending (Ia), and this physical constraint would prevent their selective activation. The target‐specific optical activation of primary afferents demonstrated by this study might provide a method to overcome these physical constraints. By refining the target specificity of the proposed method by using a cell type‐specific promoter, selective photo‐activation might be utilized in the near future.

In this study, we specifically demonstrated the optical activation of fast‐conducting, large‐diameter afferent fibres, with minimal effects on slow‐conducting fibres, by means of a recombinant AAV vector. As with CNS neurons (Galvan *et al*. 2017; Rost *et al*. 2017), this method allows the selective manipulation, not only activation, of primary sensory neurons. In the future, by employing inhibitory opsin (halorhodopsin) that pumps chloride ions into neurons and hyperpolarizes the membrane potential (Iyer *et al*. 2014; Rost *et al*. 2017), we will attempt to selectively suppress large‐diameter afferent fibres under normal behavioural conditions at millisecond levels. This will be useful for examining causal relationships between somatosensory afferent inputs and neuronal responses in the CNS as well as behavioural outcomes because this precise and rapidly reversible effect cannot be attained by electrical stimulation (Avendano‐Coy *et al*. 2018).

To date, little attention has been paid to gene therapy of large‐diameter afferents. The selective manipulation of tactile and proprioceptive afferents has clinical relevance; for example, increasing sensory flow for neuropathy and sensory ataxia and decreasing sensory input to prevent triggering spasticity in patients with stroke or spinal cord injury.

This study had some limitations. We did not use controls with AAV9 carrying an irrelevant gene. However, we tested the effects of various wavelengths of LED light (blue, green or yellow) on the ChR2‐expressing DRG neurons in all rats, and confirmed that the response was induced only when DRG neurons were exposed to the peak response spectra of ChR2 (blue light) (Fig. 2 *E*). These findings suggest that the observed effects were caused by the activation of ChR2, but not by the light stimulation itself or the effects of AAV9.

### Conclusions

We successfully activated ChR2‐expressing DRG neurons with fast‐conducting, cutaneous and proprioceptive afferents *in vivo*, and found that their response properties could be modulated by changing the pulse duration and intensity of light stimulation. Because the intervention using optogenetics suppressed or facilitated the activity of DRG neurons with high selectivity, this technique has a clear advantage over electrical stimulation for basic neuroscience and therapeutic applications.

## Additional information

### Competing interests

The authors declare that they have no competing interests.

### Author contributions

S.K. and W.S. equivalently contributed to this project. S.K. and W.S. performed the injection surgery and physiological experiments. W.S. and M.K. performed histological analysis and S.K. and T.U. performed analysis of physiological experiments. K.I. and M.T. developed virus vectors. S.K. generated the draft, and S.K. and K.S. made the final version of this paper. K.S. conceived and designed the work, and was responsible for the experiment, analysis and interpretation of data, as well as manuscript drafting, editing and revising. All authors contributed to generating the draft and final version of this paper. All authors approved the final version of the manuscript submitted for publication, and agree to be accountable for all aspects of the work in ensuring that questions related to the accuracy or integrity of any part of the work are appropriately investigated and resolved. All persons designated as authors qualify for authorship, and all qualified authors are listed. All experiment were carried out at the National Institute of Neuroscience.

### Funding

This work was supported by Grant‐in‐Aid from the Japan Society for the Promotion of Science (JSPS), Grant numbers JP26250013 (to K.S.) and JP17J05310 (to S.K.), and by research grants from the Japan Agency for Medical Research and Development (JP18dm0307021 to K.I. and JP18dm0207003 to M.T.). K.S. and K.I. were funded by the JST Precursory Research for Embryonic Science and Technology Program. S.K. was supported as a Research Fellow of the JSPS.
